# Optogenetic activation of VGLUT2-expressing excitatory neurons blocks epileptic seizure-like activity in the mouse entorhinal cortex

**DOI:** 10.1038/srep43230

**Published:** 2017-02-23

**Authors:** Latefa Yekhlef, Gian Luca Breschi, Stefano Taverna

**Affiliations:** 1 Division of Neuroscience, San Raffaele Scientific Institute, via Olgettina 58, 20132 Milan, Italy; 2 Center for Synaptic Neuroscience, Istituto Italiano di Tecnologia, via Morego 30, 16163 Genoa, Italy

## Abstract

We investigated whether an anti-epileptic effect is obtained by selectively activating excitatory neurons expressing ChR2 under the promoter for the synaptic vesicular glutamate transporter 2 (VGLUT2). VGLUT2-expressing cells were optically stimulated while local field potential and whole-cell patch-clamp recordings were performed in mouse entorhinal cortical slices perfused with the proconvulsive compound 4-aminopyridine (4-AP). In control conditions, blue light flashes directly depolarized the majority of putative glutamatergic cells, which in turn synaptically excited GABAergic interneurons. During bath perfusion with 4-AP, photostimuli triggered a fast EPSP-IPSP sequence which was often followed by tonic-clonic seizure-like activity closely resembling spontaneous ictal discharges. The GABA_A_-receptor antagonist gabazine blocked the progression of both light-induced and spontaneous seizures. Surprisingly, prolonged photostimuli delivered during ongoing seizures caused a robust interruption of synchronous discharges. Such break was correlated with a membrane potential depolarization block in principal cells, while putative GABAergic interneurons changed their firing activity from a burst-like to an irregular single-spike pattern. These data suggest that photostimulation of glutamatergic neurons triggers seizure-like activity only in the presence of an intact GABAergic transmission and that selectively activating the same glutamatergic cells robustly interrupts ongoing seizures by inducing a strong depolarization block, resulting in the disruption of paroxysmal burst-like firing.

The mechanisms responsible for the genesis and progression of epileptic seizures are not well understood. Recently, optogenetic techniques have increasingly been employed to achieve a more precise dissection of neuronal microcircuits involved in seizure generation, and to attempt to block or attenuate their progression^1^,^2^,^3^,^4^,^5^,^6^,^7^,^8^,^9^. Notably, optical stimulation of GABAergic interneurons (INs) during extracellular perfusion with 4-aminopyridine (4-AP) has been reported to trigger electrographic seizure-like events (SLEs) in slices of the mouse medial entorhinal cortex (mEC)^10^,^11^,^12^,^13^, a region highly prone to epileptic seizures in humans^14^. Parvalbumin (PV)- and somatostatin (SOM)-expressing INs displayed a similar ability to start SLEs, with modalities that closely resembled spontaneously-occurring epileptiform activity during 4-AP perfusion^10^. The onset of ictal discharges was consistently marked by a prominent raise in the extracellular concentration of potassium ions, promoting strong membrane potential depolarization and firing synchronization. In contrast with their ability to trigger SLEs, INs were insensitive to photostimulation delivered during *ongoing* tonic-clonic epileptiform activity. While selective activation of GABAergic INs should—in principle—control an ongoing seizure by means of synaptic inhibition, intracellular accumulation of Cl^−^ ions is likely to occur during strongly synchronous activity, resulting in a deleterious excitatory effect of GABA^15^,^16^,^17^,^18^. Supporting this hypothesis, archaerhodopsin-mediated selective silencing of PV-INs attenuated the occurrence of afterdischarges during the clonic phase in hippocampal slices^19^.

Given the unsuitability of INs to inhibit epileptiform activity in the 4-AP-perfused mEC, we asked whether photostimulation of glutamatergic neurons during SLEs might interfere with the synchronous burst-like firing in these cells and therefore interrupt tonic-clonic discharges. Channelrhodopsin-2 (ChR2) was expressed under the synaptic vesicular glutamate transporter 2 (VGLUT2), a protein which is highly expressed in the EC^20^. VGLUT2-expressing glutamatergic principal cells (PCs) were optically activated during perfusion with 4-AP (100–200 μM). Blue light flashes consistently triggered SLEs in these conditions—but not when GABA_A_ receptors were pharmacologically blocked. Strikingly, photostimulation of VGLUT2 neurons robustly interrupted ongoing tonic-clonic activity as PC firing underwent a strong depolarization block throughout the duration of the stimuli. During this time interneuronal firing switched from a highly synchronous rhythmic to an irregular single-spike pattern. This outcome was surprisingly much stronger than the ineffective photostimulation of PV- and SOM-INs that we had previously reported under the same conditions^10^. We suggest that activation of ChR2 in glutamatergic—rather than GABAergic—cells readily blocks ongoing seizures.

## Results

### VGLUT2-ChR2 neurons are reliably activated by optical stimuli

**Figure 1:**
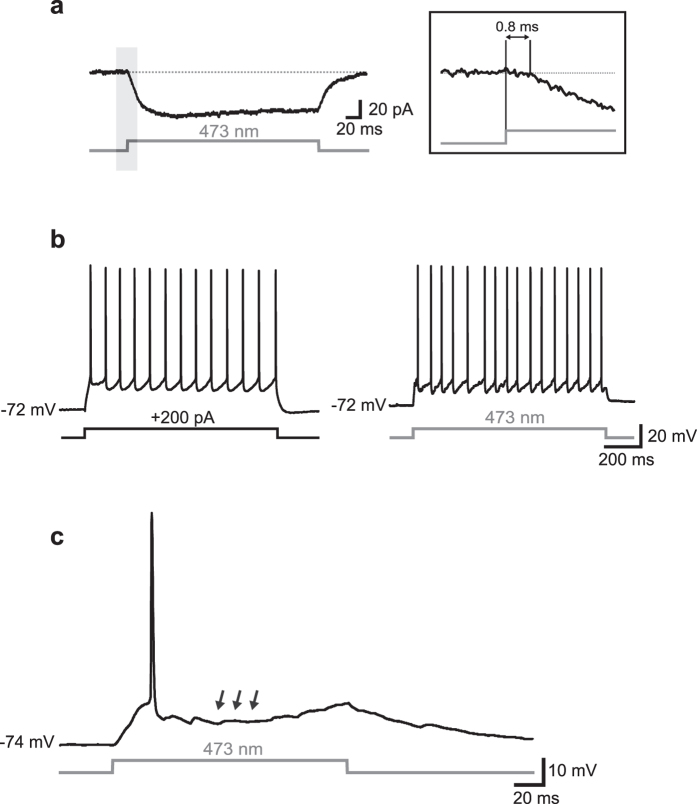
Optical activation of VGLUT2 neurons. (**a**) Example of inward current evoked by a pulse of blue light (473 nm, 250 ms) in a putative PC in the presence of synaptic blockers (5 μM NBQX, 10 μM gabazine, and 100 μM D-AP5) at V_hold_ = −80 mV. The inset shows a magnification of the trace segment included in the shaded area. Note the short latency (0.8 ms) between the flash onset and the beginning of the current deflection. **(b)** Trains of APs elicited in a different cell by injection of suprathreshold current stimulus (+200 pA, 1 s, left panel) and a photostimulus of the same length (*right*). **(c)** In a different cell, photostimulation evoked one single spike followed by a pronounced V_m_ hyperpolarization (arrows).

**Figure 2:**
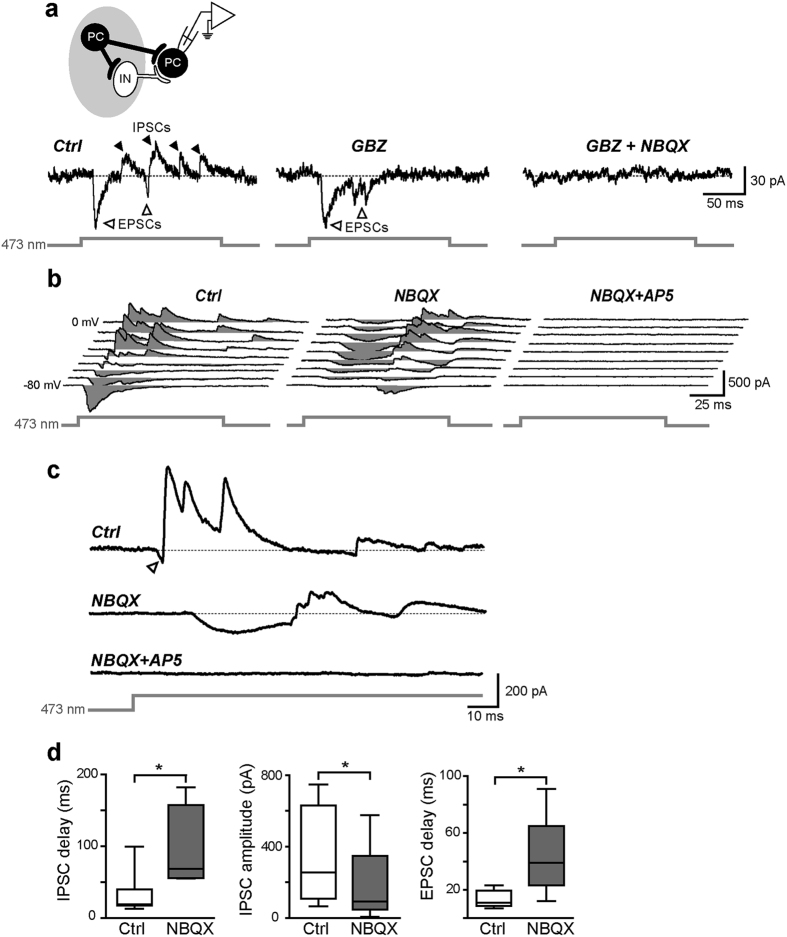
Mono- and polysynaptic excitatory and inhibitory responses evoked by VGLUT2 PC photostimulation. **(a)** Synaptic responses consisting of an EPSC-IPSC sequence elicited in a PC (recorded in voltage-clamp mode at a V_hold_ of −40 mV) by 150-ms photostimuli in control conditions (*left*) and after perfusion with 10 μM gabazine (GBZ, *middle*) followed by 5 μM NBQX (*right*). The drawing on top shows a schematic representation of the laser stimulus (grey spot) targeting presynaptic PCs (black) and interneurons (white), but not the recorded PC. Note that only PCs responded directly to light as INs did not express ChR2 (see Fig. 3). (**b**) Synaptic currents recorded in voltage-clamp mode at various command potentials (from −80 mV to 0 mV in 10-mV steps) in response to 100-ms light stimuli targeting VGLUT2-ChR2 expressing PCs. *Left*, in control conditions, recorded PCs displayed EPSC-IPSC sequences at a relatively short latency from the stimulus onset. *Middle*, blocking AMPA-receptors with 5 μM NBQX unmasked NMDA-mediated EPSCs followed by GABAergic IPSCs at longer latencies. *Right*, blockade of NMDA receptors by extracellular perfusion with D-AP5 (100 μM) completely inhibited all synaptic responses including IPSCs. **(c)** Magnification of a trace portion recorded at −30 mV shown in b. The arrowhead marks an early-onset EPSC_AMPA_ quickly curtailed by a sequence of relatively large IPSCs. **(d)** Summary box plots showing significant differences in EPSC and IPSC delays and amplitudes in control conditions and after NBQX perfusion (*p < 0.05).

Principal glutamatergic cells (PCs) and GABAergic interneurons (INs) of layers II-III of the mEC were tested for their responsiveness to blue laser optical stimuli (473 nm; Fig. 1). In voltage-clamp recordings, using a combination of synaptic antagonists (5 μM NBQX, 10 μM gabazine, and 100 μM D-AP5 to block AMPA/kainate, GABA_A_, and NMDA receptors, respectively), a large majority of VGLUT2 PCs (64 out of 83, 77%) responded to optical stimuli with a relatively steady inward current (mean amplitude and latency: −48 ± 5 pA and 0.8 ± 0.06 ms, respectively; Fig. 1a). During current-clamp recordings in plain ACSF (i.e., in the absence of synaptic blockers), photostimulation induced either a relatively regular firing activity similar to that elicited by injection of suprathreshold current steps (100–400 pA, 1 s, Fig. 1b) or just 1–2 spikes followed by a pronounced hyperpolarization (Fig. 1c). The latter phenomenon was likely due to a feedforward and/or feedback inhibition induced by a disynaptic recruitment of GABAergic INs^21^,^22^,^23^,^24^. To better assess this, we performed voltage-clamp experiments in which photostimulation of VGLUT2 cells elicited an excitatory-inhibitory postsynaptic current (EPSC-IPSC) sequence in individual PCs recorded at a V_hold_ of −40 mV, an intermediate value between the reversal potential for glutamate (0 mV) and GABA (−60 mV) in our experimental conditions (Fig. 2A; in this case the laser beam was centered ~400 μm away from the recorded cell in order to avoid eliciting a direct photocurrent which may interfere with PSCs). Extracellular perfusion with 10 μM gabazine abolished the disynaptically-evoked IPSCs; the remaining EPSC was subsequently blocked by extracellular application of NBQX (5 μM).

In the example shown in Fig. 2a, no NMDA-dependent current was visible after NBQX and GBZ perfusion, however the majority of cells (9 out of 12, 75%) displayed a robust NMDA component (Fig. 2b,c). In the attempt to confirm the polysynaptic nature of GABAergic IPSCs (and consequently rule out that they were mediated by a spurious direct photoactivation of presynaptic INs), we added 5 μM NBQX to the ACSF in order to block the EPSC-IPSC sequence. Interestingly, the IPSC arrays were not abolished, being instead evoked at a significantly later time during the optical stimulus (47 ± 13 ms in control *vs*. 96 ± 23 ms in NBQX, respectively, n = 6, p < 0.05, paired t test; Fig. 2d). The IPSC maximal peak amplitude was also significantly smaller after NBQX perfusion than in control conditions (179 ± 101 pA *vs*. 346 ± 125 pA, respectively, n = 6, p < 0.05, paired t test). Furthermore, AMPA/kainate receptor blockade by NBQX unmasked relatively slow NMDA-dependent EPSCs (EPSC_NMDA_, Fig. 2b,c) shortly preceding the GABAergic IPSCs. EPSC_NMDA_-IPSC sequences occurred at a significantly longer delay compared to EPSC_AMPA_-IPSC ones (43 ± 13 ms *vs*. 16 ± 3 ms, respectively, n = 6, p < 0.05, Wilcoxon signed-rank test). Subsequent block of NMDA receptors by bath application of 100 μM D-AP5 completely eliminated both inward and outward currents (Fig. 2b,c). Altogether, these data suggest that glutamate release caused by photostimulation of VGLUT2 neurons elicits monosynaptic AMPA/kainate- and NMDA-mediated ESPCs on both glutamatergic neurons and GABAergic interneurons; the latter cells in turn project inhibitory synaptic contacts onto glutamatergic neurons. Interestingly, the presence of polysynaptic IPSCs during NBQX perfusion—and their complete inhibition during D-AP5 application—indicates that NMDA receptors alone, despite using slower kinetics than AMPA receptors, are able to produce a suprathreshold response and trigger action potentials (APs) in interneurons^25^.

**Figure 3:**
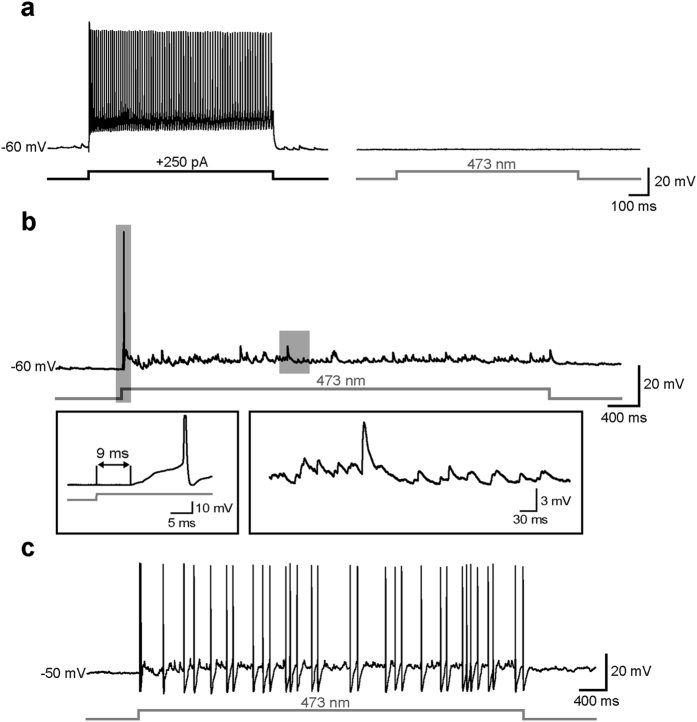
Interneurons are synaptically but not directly activated by optical stimuli. **(a)** Left, example of high-frequency firing activity evoked in a putative IN in response to injection a suprathreshold current stimulus (250 pA, 1 s). Right, a 1-s light stimulus directly centered on the same interneuron in the presence of 5 μM NBQX and 10 μM GBZ did not evoke any significant response. **(b)** Representative trace showing synaptic activity elicited in a putative IN in response to a photostimulus in the absence of synaptic blockers. Shaded rectangles include portions of the trace magnified in bottom insets. The first depolarizing deflection of V_m_ (shown in left inset) occurred at a relatively high latency from the stimulus onset (9 ms), triggering one occasional early spike. An array of postsynaptic potentials (PSPs, magnified in right inset) were elicited throughout the remainder of the stimulus. **(c)** When the flash was delivered at a more depolarized V_m_ (−50 mV), the synaptic response was sufficient to reach the firing threshold, resulting in robust spiking activity.

To assess the specificity of photostimulation we also recorded putative fast-spiking INs (n = 19, Fig. 3), which were characterized by a firing activity at relatively high frequencies (90 ± 4 Hz). Direct illumination of both types of INs with blue light evoked no ChR2-dependent response in the presence of synaptic blockers (Fig. 3a), confirming that ChR2 was specifically expressed in PCs. However, in plain ACSF a robust array of postsynaptic potentials (PSPs) was elicited throughout the length of the optical stimulus, occasionally triggering 1–2 spikes when cells were held at a relatively hyperpolarized potential (e.g. −60 mV, Fig. 3b). At more depolarized potentials (>−55 mV) the PSPs often reached the threshold for firing, resulting in sustained spike trains (Fig. 3c). The latency of light-induced depolarization was significantly higher in INs than in PCs (7 ± 0.5 ms *vs*. 1 ± 0.1 ms, n = 21 and 64, respectively, Mann-Whitney rank sum test, p < 0.001), confirming that INs were not directly driven by ChR2-mediated currents (as they do not express VGLUT2, consistently with their GABAergic nature) but were synaptically activated by photostimulation of presynaptic glutamatergic cells. Similar results were obtained in putative somatostatin-expressing INs (n = 4, not shown), which were identified by post-anodal rebound firing and a prominent depolarizing sag in response to injection of hyperpolarizing current pulses^10^.

### Photoactivation of VGLUT2 neurons triggers tonic-clonic seizures through recruitment of GABAergic interneurons

**Figure 4:**
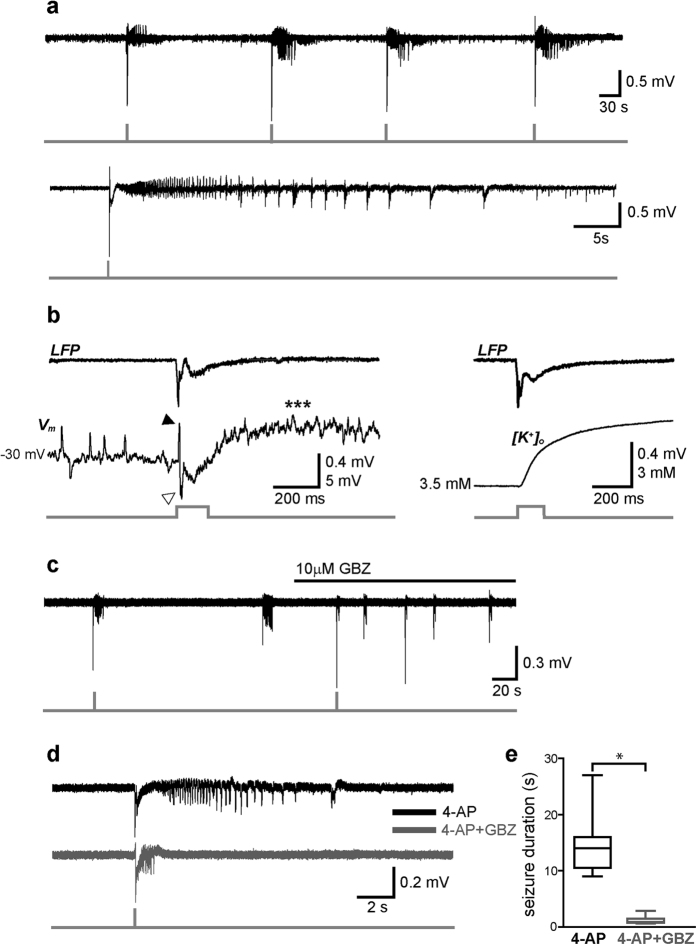
Optical activation of VGLUT2 neurons triggers GABA_A_-receptor-dependent SLEs. **(a)** Top, example of LFP recording showing four SLEs evoked by brief photostimuli (150 ms) during extracellular perfusion with 4-AP. Bottom, magnification of an individual SLE characterized by a flash-evoked large spike followed by a typical tonic-clonic pattern. **(b)** *Left*, close-up of the initial stage of a SLE showing a flash-evoked spike (LFP trace on top) corresponding to an EPSP-IPSP sequence (black and white arrowheads, respectively, in simultaneous whole-cell current-clamp trace on bottom) followed by a depolarizing wave (asterisks). The initial V_m_ was maintained at −30 mV by steady current injection (+400 pA). Note upward- and downward-deflecting EPSPs and IPSPs, respectively, preceding the flash. *Right*, in a different recording, the flash-evoked spike was followed by a rise in extracellular potassium concentration ([K^+^]_o_). **(c)** Both spontaneous and light-triggered SLEs were strongly shortened during extracellular perfusion with the GABA_A_ receptor antagonist gabazine (GBZ). **(d)** Magnified SLE traces in control condition (black) and during perfusion with GBZ (grey). **(e)** Box-plot summarizing average SLE lengths before and after blocking GABA_A_ receptors with GBZ (*p < 0.05).

To test whether selective activation of VGLUT2 neurons triggers seizure-like events (SLEs), slices were extracellularly perfused with ACSF containing the proconvulsive compound 4-aminopyridine (4-AP, 100–200 μM). After 12–15 min from the beginning of the perfusion, photostimulation of VGLUT2 neurons reliably induced SLEs at a brief latency (2 ± 0.13 ms, measured as the interval between stimulus onset and the initial large spike onset; Fig. 4a). In 7 slices out of 8 (87%), 200-ms light flashes evoked fast EPSP-IPSP sequences followed by a slower depolarizing phase, which shortly preceded the SLE and was associated with an increase in the concentration of extracellular potassium ([K^+^]_o_) (Fig. 4b).

Using optogenetic tools, we and others have recently reported that seizure initiation is to be attributed to an abnormal activation of GABAergic interneurons^10^,^11^. Here, SLEs were also elicited by selectively stimulating glutamatergic cells, however blockade of GABA_A_ receptors during bath application of 10 μM gabazine strongly reduced the length of flash-evoked SLEs (ctrl: 18 ± 4 s, GBZ: 2.4 ± 0.2 s, p < 0.001, n = 13, Wilcoxon signed rank test; Fig. 4c–e), suggesting that photoactivation of glutamatergic cells alone is not sufficient to trigger tonic-clonic seizures without the recruitment of an intact interneuron-mediated GABAergic transmission.

### Photostimulation of VGLUT2-expressing neurons during ongoing SLEs interrupts ictal discharges

**Figure 5:**
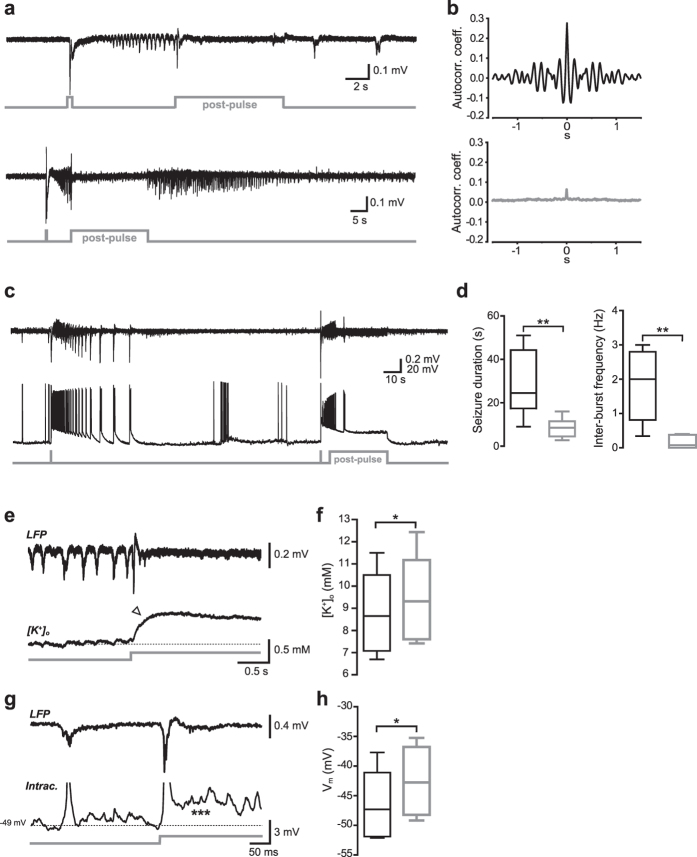
Optical activation of VGLUT2 neurons blocks ongoing SLEs. **(a)** Two examples of LFP recordings in which blue light (“post-pulse”, 8 and 20 s, respectively) was turned on during ongoing SLEs, causing the interruption of synchronous clonic discharges throughout the stimuli. **(b)** Representative autocorrelograms computed for trace portions before (top) and during (bottom) the post-pulse. The small residual peak at t = 0 in the bottom plot represents noise autocorrelation. **(c)** In another example of post-pulse-induced seizure block, a simultaneous current-clamp recording from an individual VGLUT2 neuron (bottom trace) shows that burst-like firing activity was strongly reduced during the post-pulse as compared to a previous control SLE without post-pulse (left portion of traces). **(d)** Summary box plots with average SLE durations (left) and inter-burst frequencies (right) in control conditions (black) and during post-pulse illumination (grey; *p < 0.05). **(e)** Excerpt of an LFP trace segment showing a post-pulse-induced SLE interruption in correspondence with a simultaneously recorded [K^+^]_o_ trace obtained with a K-selective electrode. The arrowhead points to an increase in [K^+^]_o_ associated with the break in clonic firing. **(f)** Summary of average [K^+^]_o_ measured prior to and during the post-pulse at the steady-state level (*p < 0.05). **(g)** Excerpt of an LFP trace segment (top) showing post-pulse-induced interruption of a SLE in correspondence with a simultaneous intracellular recording (bottom) in a VGLUT2 neuron. The asterisks indicate a transient V_m_ depolarization following the post-flash onset (spikes have been truncated for clarity). **(h)** Summary of average V_m_ measured prior to and during the post-pulse at the steady-state level (*p < 0.05).

Recent data from our group showed that selective activation of either SOM- or PV-interneurons was unable to stop hypersynchronous discharges when light stimuli were delivered during ongoing SLEs^10^. Contrary to the unresponsiveness of interneurons, here photostimulation of VGLUT2 neurons induced a robust block of synchronous discharges (Fig. 5). In most experiments (20 out of 26 slices, 77%), photostimuli (“post-pulses”) lasting 5–35 s and delivered at different stages of the tonic-clonic phase (during either spontaneous or light-induced SLEs) strongly reduced or completely blocked hypersynchronous LFP spikes (Fig. 5a). Consistent with a substantial loss of rhythmic discharging, autocorrelation analysis of LFP trace portions recorded during post-pulses resulted in relatively flat plots, compared to multi-peaked autocorrelograms corresponding to trace portions immediately preceding the flashes (Fig. 5b). Interestingly, when the post-pulse duration was shorter than the length of control SLEs the block would only be temporary (i.e., the SLE quickly resumed after the pulse offset; Fig. 5a). On the other hand, prolonged pulses (20–30 s) resulted in a significant reduction in SLE duration (ctrl: 33 ± 4 s, post-pulse: 11 ± 1 s, n = 19, p < 0.01, Wilcoxon signed rank test; Fig. 5c,d).

Simultaneous whole-cell recordings showed that individual PCs—which during SLEs fired AP bursts highly synchronous with the tonic-clonic discharges—underwent a robust depolarization block for the duration of the light stimulus (Fig. 5c). Such a block would occur either immediately or within a short delay (up to 4–5 seconds) after the post-stimulus onset. The mean inter-burst frequency during the post-pulse was significantly reduced compared to intact SLEs (0.16 ± 0.07 Hz *vs*. 1.9 ± 0.4 Hz, respectively, n = 6, p < 0.05, paired t test; Fig. 5d).

### The post-pulse-induced depolarization block is accompanied by an increase in extracellular K^+^ concentration

The strong reduction in PC firing activity appeared to be due to a V_m_ depolarizing block, which was accompanied by a small yet significant increase in [K^+^]_o_ (mean concentration before post-pulse: 9.3 ± 0.9 mM, during post-pulse: 10.0 ± 0.9 mM, p < 0.05, n = 5, paired t test; Fig. 5e,f). This change was likely induced by the transient firing episode in response to the flash, and possibly sustained by K^+^ efflux through ChR2 channels during photoactivation. The small raise was associated with a transient depolarization (V_m_ before post-pulse: −47 ± 6 mV, during post-pulse: −42 ± 6 mV, p < 0.05, n = 6, paired t test; Fig. 5g,h). We asked whether the increase in [K^+^]_o_ was effective in blocking further synchronous firing in PCs, as ChR2-mediated inward Na^+^ current may, in principle, be sufficient to cause V_m_ depolarization and block cell firing. To test the roles of ChR2 inward current *vs.* locally increased extracellular K^+^, we injected a small current step (100–200 pA, 5–10 s) into an individual cell shortly after an SLE was started (Supplementary Fig. S1). The current step amplitude was chosen based on the steady-state amplitude of a flash-induced I_ChR_ previously recorded in the same cell (inset in panel a). During the current step injection, a puff of ACSF containing 8–10 mM KCl was briefly delivered for 5–10 s onto the cell using a local perfusion pipette. The firing rate decreased from 7.5 ± 1.3 Hz to 5.4 ± 1.2 Hz (n = 4, p > 0.05, paired t test) during current injection alone, but was even further reduced—or ceased—during the subsequent KCl puff (mean frequency 1.1 ± 0.5 Hz, n = 4, p < 0.05, paired t test; panels b and c). This suggests that the increase in [K^+^]_o_ associated with ChR2 activation is required for blocking cell firing during VGLUT2 photostimulation.

### Interneuronal burst firing is blocked during the post-pulse

**Figure 6:**
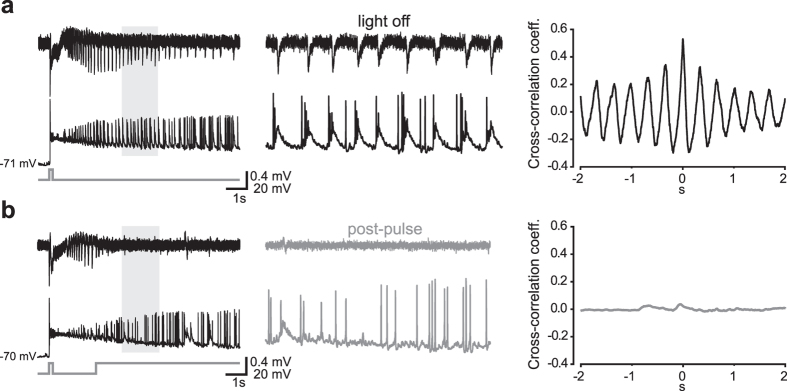
Synchronous burst-like firing activity in interneurons is abolished by optical activation of VGLUT2 neurons during SLEs. **(a)** LFP (top) and whole-cell current-clamp (bottom) simultaneous recordings of a control SLE triggered by a 150-ms photostimulus. Trace portions included in the shaded area are magnified in the middle panel. *Right*, cross-correlogram showing temporal association between the cell burst-like firing activity and LFP synchronous spikes. **(b)** During a subsequent SLE, a post-pulse delivered shortly after the SLE onset interrupted the synchronous discharges. The IN burst-like activity turned into sparse AP firing (*middle*) while the correlation with the LFP activity was lost (*right*).

**Figure 7:**
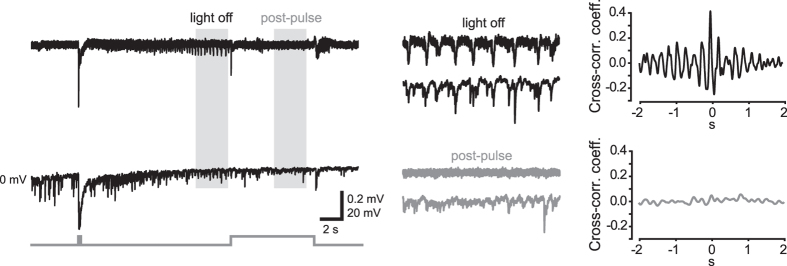
Optical activation of VGLUT2 neurons disrupts synchronous IPSC arrays in PCs. LFP (top) and whole-cell current-clamp (bottom) simultaneous recordings of a control SLE triggered by a 150-ms photostimulus. Trace portions included in the shaded areas are magnified in middle panels. *Right*, cross-correlograms showing temporal association between IPSC arrays and LFP synchronous spikes prior to and during an 8-s lasting post-pulse.

During the post-pulse, GABAergic interneurons switched their firing pattern from a highly bursting to a sparsely single-spike mode (n = 6 out of 6 INs, 100%; Fig. 6), likely because the depolarization block in VGLUT2 neurons greatly reduced synaptic excitation onto these cells. Accordingly, IPSP bursts recorded at 0 mV in PCs in correspondence with synchronous LFP spikes were abolished or reduced during post-pulses (n = 5 out of 7 PCs, 71%; Fig. 7). In control SLEs, these repetitive IPSP bursts may cause an excessive accumulation of intracellular chloride in postsynaptic cells, thus playing a critical role in paroxysmal firing by causing a switch from inhibitory to excitatory GABAergic transmission^15^,^16^,^17^. Hence, their disruption during post-pulses additionally contributed to silencing principal cells.

### VGLUT2 cell photostimulation controls epileptiform activity in the low-Mg^2+^/high K^+^ model

Finally, we tested another model that is able to induce epileptiform activity by perfusing slices with ACSF containing 0.25 mM MgCl_2_ and 8 mM KCl^26^. In these conditions seizure-like discharges were also blocked by photostimulating VGLUT2 neurons (ctrl duration: 57 ± 13 s, post-pulse: 34 ± 13 s, n = 4, p < 0.05, Wilcoxon signed rank test; Supplementary Fig. S2), indicating that the inhibitory effect of VGLUT2 neuron activation was not specific to the 4-AP model of seizure induction.

In summary, these data suggest that photostimulation of VGLUT2 neurons during ongoing seizures: (1) causes a robust depolarizing block in these cells; (2) reduces their strong, synchronous excitatory drive to interneurons; and (3) blocks paroxysmal tonic-clonic discharges and interrupts the seizure.

## Discussion

In this study optogenetic stimulation of principal glutamatergic cells during perfusion with 4-AP resulted in a high probability of induced tonic-clonic SLEs in mouse medial entorhinal cortex slices. Such epileptiform activity was strongly reduced in the presence of a GABA_A_-receptor blocker, indicating that SLEs require an intact synaptic GABAergic transmission and cannot be triggered by PC photostimulation alone. Most importantly, PC photostimulation delivered during ongoing SLEs robustly interrupted or terminated the synchronous discharges. Concurrently recorded individual PCs displayed an effective depolarizing block during the photostimulation, while putative interneurons diminished their contribution to paroxysmal discharges by switching from a bursting to an irregularly single-spike pattern.

The large majority of putative glutamatergic cells recorded in layer II-III of the mEC directly responded to blue light illumination, in line with a robust expression of VGLUT2^20^. Conversely, no direct ChR2-dependent current was detected in putative GABAergic interneurons in the presence of excitatory and inhibitory synaptic receptor blockers, confirming the specificity of ChR2 expression. In these cells, however, photostimulation of VGLUT2 neurons in plain ACSF (i.e., without synaptic blockers) elicited trains of EPSPs which readily induced AP firing when V_m_ was close to threshold. Interestingly, synaptic recruitment of interneurons was possible even in the presence of the AMPA/kainate receptor antagonist NBQX, suggesting that the depolarization induced by activation of NMDA receptors alone was sufficient to trigger spike firing in these cells, as previously reported using electrical stimulation of EC deep layers^25^.

Recent *in vitro* studies have shown that SLEs are triggered by synchronous activation of GABAergic interneurons^10^,^11^,^12^,^27^,^28^,^29^. Here, optical activation of VGLUT2 neurons also elicited tonic-clonic SLEs during extracellular perfusion with 4-AP, however even in these conditions interneuron-mediated GABAergic transmission appeared to play a critical role. The initial stage of seizures was indeed marked by an EPSP-IPSP sequence, followed by a relatively slow depolarizing wave associated with an increase in extracellular K^+^ (Fig. 4B), possibly caused by the activation of the K^+^-Cl^−^ cotransporter KCC2^30^. Both spontaneous and photo-induced SLEs were blocked in the presence of gabazine, confirming that GABA_A_ receptor activation is necessary for this type of epileptiform activity in the mEC.

Strikingly, VGLUT2 neuron photostimulation consistently arrested the ictal discharge when delivered during the tonic-clonic phase of an ongoing SLE. This effect may seem at odds with the strong propensity of the same cells to induce SLEs, however the timing of the stimulation appeared to be critical as the excitability state of a given neuron—and therefore its electrical responsiveness to perturbations—is clearly different depending on whether the cell membrane potential is hyperpolarized (i.e., near the resting level), as opposed to oscillating in a burst-like firing mode typical of tonic-clonic discharges. In the hyperpolarized state, ChR2-induced firing activity generated a relatively large extracellular K^+^ wave resulting in a strong V_m_ depolarization and a progressively increasing involvement of neurons in burst-like firing activity^31^,^32^,^33^,^34^,^35^. The delivery of another photostimulus at this stage elicited a further, yet smaller (due to a reduced driving force), [K^+^]_o_ wave. This caused some further V_m_ depolarization which, although relatively small and partially decremental with time, was sufficient to substantially block spike firing and interrupt or terminate the SLE.

During the delivery of post-pulses we observed a disruption of synchronous interneuronal discharges, along with a reduction of IPSC arrays recorded in PCs (Figs 6 and 7). This effect may actually help stop the SLE, as synchronous arrays of IPSCs likely cause an excessive accumulation of intracellular chloride, leading to a switch in the reversal potential for that ion and transforming GABAergic transmission from inhibitory to excitatory^15^,^16^,^17^. During post-flashes, interneuronal firing turned from a synchronous bursting to a sparse single-spike pattern, while strong IPSP arrays were substituted by individual IPSPs. This may have helped reduce the intracellular Cl^−^ load, restoring a normal inhibitory GABAergic function.

Interestingly, epileptiform activity was spontaneously resumed at the end of relatively brief post-pulses (Fig. 5a; see also Fig. 10C in ref. 10). This ability to continue temporarily affected synchronous discharges suggests that 4-AP-dependent SLEs correspond to very robust hypersynchronous patterns which, even when briefly interrupted, will carry on until they reach their natural endpoint. Therefore, relatively long-lasting post-pulses were needed in order to maintain the interruption for enough time to actually stop the SLE (Fig. 5c,d).

It is not completely clear why ChR2-expressing PCs were so much more prone to undergo a photostimulation-driven depolarization block than ChR2-expressing PV- or SOM-interneurons in the same conditions^10^. It is possible that different combinations of active ionic conductances that shape membrane excitability during heavy paroxysmal burst-like firing cause these cell types to respond differently to further ChR2-mediated depolarization. In addition, stimulation of PCs (which largely outnumber INs) may induce a critical increase in [K^+^]_o_ which perhaps could not be achieved when interneurons alone were activated. In any case, stimulating INs during seizures may actually result in a negative outcome by yielding an excitatory rather than inhibitory effect^19^. This is probably due to a collapse of the transmembrane Cl^−^ gradient, and therefore a depolarizing shift of the GABA_A_ reversal potential^19^,^36^,^37^ (but see. refs 2 and 38). Thus, photostimulation of glutamatergic cells, with the aim to stop their firing and prevent glutamatergic activation of interneurons, seems to be a preferable strategy to block SLEs-at least in the mEC-in our experimental conditions.

In conclusion, we show that optogenetic stimulation of glutamatergic principal cells in the mEC triggers tonic-clonic SLEs which are dependent on synaptic activation of GABAergic interneurons and a functional GABAergic transmission. Further to this, however, photostimulation of the same cells during ongoing SLEs caused a robust interruption of the synchronous discharges, thanks to a depolarization block of PCs and a consequently reduced synaptic activation of interneurons.

In future *in vivo* studies, light-induced stimulation or inhibition of specific cell populations will suggest effective therapeutic interventions, which could be selectively designed and customized across both different affected brain areas and diverse patterns of pathological hyperexcitability^1^,^3^,^4^,39,^40^.

## Methods

### Slice preparation and electrophysiology

Recordings were performed in horizontal compound entorhinal-hippocampal slices prepared from a recombinant Cre-Lox mouse line obtained by crossing B6.Cg-Gt(ROSA)26Sortm27.1(CAG-COP4*H134R/tdTomato)Hze/J mice (JAX stock number: 012567; Jackson Laboratory, Bar Harbor, ME, USA) with Slc17a6tm2(cre)Lowl (JAX stock number: 016963). The offspring, which appeared viable and healthy, selectively expressed ChR2 in VGLUT2-expressing cells. All procedures were approved by the Italian Ministry of Health and the San Raffaele Scientific Institute Animal Care and Use Committee in accordance with the relevant guidelines and regulations. Mice of both sexes (30–50 days of age) were anesthetized with an intraperitoneal injection of a mixture of ketamine/xylazine (100 mg/kg and 10 mg/kg, respectively) and perfused transcardially with ice-cold artificial cerebrospinal fluid (ACSF) containing (in mM): 125 NaCl, 3.5 KCl, 1.25 NaH_2_PO_4_, 2 CaCl_2_, 25 NaHCO_3_, 1 MgCl_2_, and 11 D-glucose, saturated with 95% O_2_ and 5% CO_2_ (pH 7.3). After decapitation, brains were removed from the skull and 300 μm-thick horizontal slices containing the entorhinal cortex and the hippocampus were cut in ACSF at 4 °C using a VT1000S vibratome (Leica Microsystems, Wetzlar, Germany). Individual slices were then submerged in a recording chamber mounted on the stage of an upright BX51WI microscope (Olympus, Japan) equipped with differential interference contrast optics (DIC). Slices were perfused with ACSF continuously flowing at a rate of 2–3 ml/min at 32 °C. Whole-cell patch-clamp recordings were performed in layer II/III of the medial entorhinal cortex (mEC) using pipettes filled with a solution containing the following (in mM): 10 NaCl, 124 KH_2_PO_4_, 10 HEPES, 0.5 EGTA, 2 MgCl_2_, 2 Na_2_-ATP, 0.02 Na-GTP, (pH 7.2, adjusted with KOH; tip resistance: 4–6 MΩ). Local field potentials (LFPs) were recorded using extracellular electrodes (tip resistance 1–2 MΩ) filled with ACSF and positioned in layers II-III of the mEC, ~250 μm below the pial surface and 100–200 μm laterally from the patch electrode. Local perfusion with a high-potassium solution was achieved by placing a glass pipette (tip diameter: 5–7 μm) close to the recorded cell at a distance of ~10 μm. Brief puffs (5–10 s) of solution were ejected using a Picospritzer III fluid dispensing system (Parker Hannifin, Hollis, NH, USA). All recordings were performed using a MultiClamp 700B amplifier interfaced with a PC through a Digidata 1440A (Molecular Devices, Sunnyvale, CA, USA). The liquid junction potential was not corrected. The series resistance was partially compensated (40–50%) using the amplifier control circuit.

### Data acquisition and analysis

Data were acquired using pClamp10 software (Molecular Devices) and analyzed with Origin 9.1 (Origin Lab, Northampton, MA, USA). Voltage- and current-clamp traces were sampled at a frequency of 30 kHz and low-pass filtered at 2 kHz. Local field potentials were acquired at a frequency of 10 kHz and band-passed within a 1 Hz-1 kHz frequency range.

### Photostimulation of ChR2-expressing neurons

Optical stimuli were generated by a diode-pumped solid state laser (wavelength: 473 nm; light power at the source: 100 mW; Shanghai Dream Lasers Technology, Shanghai, China) connected to the epi-illumination port of the microscope through a multi-mode optical fiber. The beam was deflected by a dichroic mirror and conveyed to the slice through a 40x water-immersion objective (spot size: 0.06 mm^2^). The light power measured with an optical power meter at the level of the slice surface was ~2 mW, yielding a light density value of ~33 mW/mm^2^. Photostimuli were TTL-triggered using Clampex digital output signals.

The latencies between optical stimuli and ChR2-induced direct or synaptic currents in patched neurons were measured as the distance between the stimulus trigger onset (recorded as a digital output trace in pClamp) and the time at which membrane potential traces deflected by twice the standard deviation (SD) of the baseline mean value measured within a 20-ms time window preceding the stimulus.

### Extracellular potassium recordings

Variations in extracellular potassium concentration ([K^+^]_o_) were recorded using standard glass electrodes (OD: 1.5 mm; ID: 0.86 mm; Harvard Apparatus, Holliston, MA, USA). Pipettes were exposed to dimethyldichlorosylane vapors (Sigma) for 1 min and then heated at 120 °C for 2 hr. After cooling at room temperature, the tips (diameter 3–5 μm) were filled with potassium ionophore I - cocktail A (Sigma), then the pipettes were back-filled with an aqueous solution containing 3 mM KCl and 150 mM NaCl^41^. Individual pipettes were mounted on a high-input impedance headstage amplifier (HS2, Molecular Devices) connected to a MultiClamp 700B amplifier. Signal calibration was obtained by dipping the tip of the ion-selective electrode in ACSF and recording voltage changes in response to switching bath solutions containing progressively increasing K^+^ concentrations (1, 2.5, 6, 12.5, and 48 mM). Voltage values were collected when plateau levels were reached after any solution exchange. The values were then fit with the linear equation y = b*log x, where x is [K+]o, y is the voltage, and b is the slope coefficient. During experiments, the K-sensitive electrode was placed laterally to the LFP-recording one at a distance of 100–200 μm.

### Statistical analysis

Datasets were compared using paired or unpaired Student’s t-tests for normally distributed datasets, and Wilcoxon signed rank or Mann-Whitney rank sum tests otherwise (SigmaStat, Systat Software, Chicago, IL). Results are given as means ± s.e.m. in text and represented by box plots in figures. Boxes include 25^th^ and 75^th^ percentiles (horizontal edges), median value (inner line), and min-max values (whiskers). Differences were considered significant at p < 0.05.

### Drugs

All drugs were obtained from Sigma except 2,3-dioxo-6-nitro-1,2,3,4-tetrahydrobenzo[*f*]quinoxaline-7-sulfonamide disodium salt (NBQX), D-(−)-2-Amino-5-phosphonopentanoic acid (D-AP5), and 2-(3-carboxypropyl)-3-amino-6-(4 methoxyphenyl)pyridazinium bromide (gabazine) which were obtained from Abcam Biochemicals (Bristol, UK).

## Additional Information

**How to cite this article:** Yekhlef, L. *et al*. Optogenetic activation of VGLUT2-expressing excitatory neurons blocks epileptic seizure-like activity in the mouse entorhinal cortex. *Sci. Rep.* **7**, 43230; doi: 10.1038/srep43230 (2017).

**Publisher's note:** Springer Nature remains neutral with regard to jurisdictional claims in published maps and institutional affiliations.

## Supplementary Material

Supplementary Information
