# Supplementary material for: Optogenetic activation of VGLUT2-expressing excitatory neurons blocks epileptic seizure-like activity in the mouse entorhinal cortex
Source: Sci Rep. 2017 Feb 23;7:43230. doi: 10.1038/srep43230 (PMC5322365; doi:10.1038/srep43230)
Supplement: Supplementary Information [file srep43230-s1.pdf]

Supplementary Information

Optogenetic activation of VGLUT2-expressing excitatory neurons blocks epileptic seizure-like activity in the mouse entorhinal cortex.

Latefa Yekhlef[1,2], Gian Luca Breschi[1], and Stefano Taverna[1,*]

[1]Division of Neuroscience, San Raffaele Scientific Institute, via Olgettina 58, 20132 Milan, Italy.

[2]Center for Synaptic Neuroscience, Istituto Italiano di Tecnologia, via Morego 30, 16163 Genoa, Italy.

*Corresponding author, taverna.stefano@hsr.it

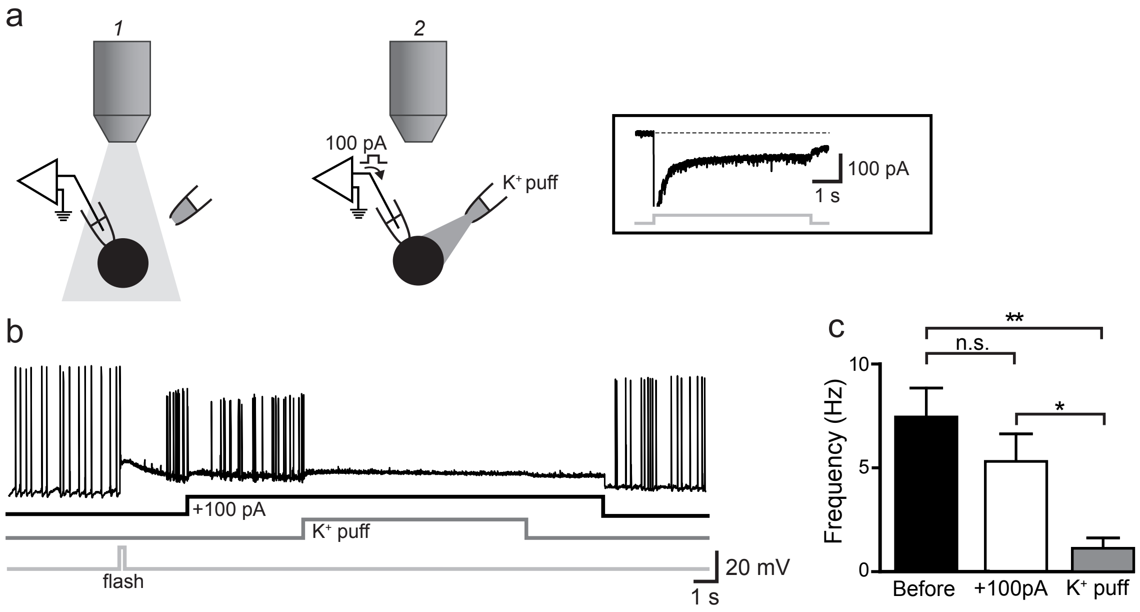

Supplementary Figure S1. Depolarization block is mimicked in individual neurons by injection of a small current pulse combined with local perfusion with exogenous extracellular potassium. (a) Schematic panel showing the experimental design. *1*, during perfusion with 4-AP, a VGLUT2 neuron was recorded while a brief laser flash was delivered through the microscope objective to induce a SLE. *2*, a rectangular current step (100 pA, 10s) was injected in the cell through the patch pipette shortly after the SLE initiation. During the pulse injection, a brief puff of ACSF containing 9 mM KCl was delivered through a second pipette positioned ~10 μm laterally from the cell. The inset shows a current trace evoked in the cell during a 5-s flash of blue light during 4-AP perfusion. The same value of the light-induced current steady-state amplitude was used for the injected current pulse (100 pA in this case). **(b)** Example of a flash-induced seizure during which a current step was injected in the cell, resulting in a reduced, but not completely inhibited, spike firing activity. During a subsequent KCl puff, AP firing was completely stopped. **(c)** Summary of average firing frequencies during a SLE immediately before the injection a current step (excluding the initial plateau potential without spikes), during the injection of the current step (+100 pA), and during the following local perfusion with 9 mM KCl (K^+ puff). (*$p < 0.05$, **$p < 0.01$).

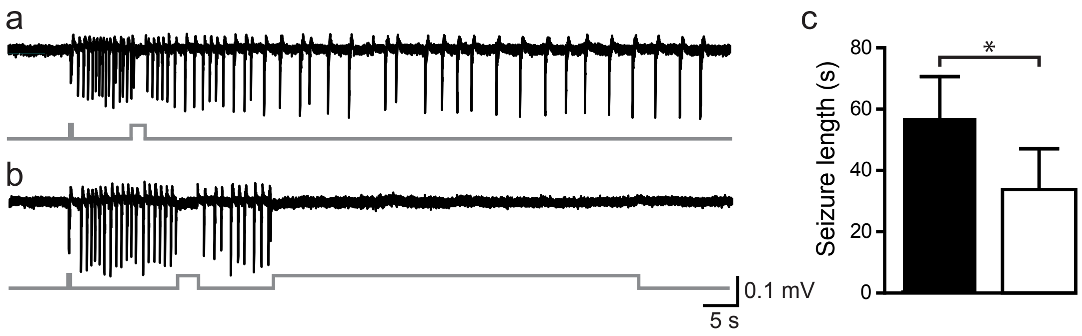

Supplementary Figure S2. Post-pulses are also effective in blocking epileptiform activity recorded in a low-magnesium model of seizures. (a) Example of SLE evoked in the mEC during extracellular perfusion with ACSF containing 0.25 $MgCl_2$ and 8 mM KCl. Note that a brief (3s) flash of blue light temporarily interrupted the synchronous discharges. **(b)** During a subsequent SLE, a longer pulse (50s) completely blocked the synchronous activity. **(c)** Summary plot of average seizure length in the absence (black) and presence (white) of a post-pulse (*$p < 0.05$).
